# RGB Three-Channel SWE-Based Ultrasomics Model: Improving the Efficiency in Differentiating Focal Liver Lesions

**DOI:** 10.3389/fonc.2021.704218

**Published:** 2021-09-27

**Authors:** Mei-Qing Cheng, Meng-Fei Xian, Wen-Shuo Tian, Ming-De Li, Hang-Tong Hu, Wei Li, Jian-Chao Zhang, Yang Huang, Xiao-Yan Xie, Ming-De Lu, Ming Kuang, Wei Wang, Si-Min Ruan, Li-Da Chen

**Affiliations:** ^1^ Department of Medical Ultrasonics, Institute of Diagnostic and Interventional Ultrasound, Ultrasomics Artificial Intelligence X-Lab, The First Affiliated Hospital of Sun Yat-Sen University, Guangzhou, China; ^2^ Department of Hepatobiliary Surgery, The First Affiliated Hospital of Sun Yat-Sen University, Guangzhou, China

**Keywords:** ultrasonography, elasticity imaging techniques, machine learning, radiomics, liver

## Abstract

**Objective:**

To explore a new method for color image analysis of ultrasomics and investigate the efficiency in differentiating focal liver lesions (FLLs) by Red, Green, and Blue (RGB) three-channel SWE-based ultrasomics model.

**Methods:**

One hundred thirty FLLs were randomly divided into training set (n = 65) and validation set (n = 65). The RGB three-channel and direct conversion methods were applied to the same color SWE images. Ultrasomics features were extracted from the preprocessing images establishing two feature data sets. The least absolute shrinkage and selection operator (LASSO) logistic regression model was applied for feature selection and model construction. Two models, named RGB model (based on RGB three-channel conversion) and direct model (based on direct conversion), were used to differentiate FLLs. The diagnosis performance of the two models was evaluated by area under the curve (AUC), calibration curves, decision curves, and net reclassification index (NRI).

**Results:**

In the validation cohort, the AUC of the direct model and RGB model in characterization on FLLs were 0.813 and 0.926, respectively (*p* = 0.038). Calibration curves and decision curves indicated that the RGB model had better calibration efficiency and provided greater clinical benefits. NRI revealed that the RGB model correctly reclassified 7% of malignant cases and 25% of benign cases compared to the direct model (*p* = 0.01).

**Conclusion:**

The RGB model generated by RGB three-channel method yielded better diagnostic efficiency than the direct model established by direct conversion method. The RGB three-channel method may be promising on ultrasomics analysis of color images in clinical application.

## Introduction

Shear-wave elastography (SWE), as an elasticity-based US technique, is widely used in lesion characterization and liver stiffness assessment (1–4). It can quantitatively evaluate tissue stiffness by measuring the velocity (m/s) of shear wave or tissue elastic modulus (kPa). However, SWE is still operator dependent in the optimal region selection, and there are few established guidelines on how to acquire a satisfied SWE image, which resulted in interobserver variability and subjective diagnostic decision-making (5, 6).

Radiomics or ultrasomics is a promising field for image analysis, through extracting a high throughput of quantitative data from medical images for clinical application (7). Its potential to provide a better insight into tumor characteristics that fail to be appreciated by naked eyes facilitates a direct estimation of outcomes (8). Recent advances related to ultrasomics involved tumor detection, classification, staging, and therapeutic assessment. In tumor classification, ultrasomics is mainly based on an analysis of the grayscale image due to the heterogeneity of the tumor itself. However, interpreting color images is also required in clinical practice such as SWE image analysis. For color image analysis, the most common existing method is directly converted into a grayscale image, which is usually done by the following color-to-grayscale algorithms: Intensity, Luminance, Lightness, Value, and so on (9). According to human’s sensitivity to the R, G, and B colors, Lightness, which is based on the formula of gray intensity = 0.2126R + 0.7152G + 0.0722B, more closely corresponds to human perception and preferable in color image conversion, which is achieved *via* a nonlinear transformation of the RGB color space. However, it is not a lossless method for color to grayscale image conversion, which suffers from a domain shifting problem due to the overlaps of Red, Green, and Blue (RGB) channel values (9, 10). Other common analysis methods included RGB to grayscale elasticity map (5, 11–14) and histogram-based color image analysis (15). The RGB to grayscale elasticity image is a lossless inverse procedure, but it required an intermediate image processing step (such as RGB to Stiffness conversion) for image analysis. The drawback of the latter method was that information about the object’s location, shape and texture are discarded.

A representative SWE image consists of a grayscale accompanied with a corresponding color SWE image. The RGB color model is composed of three primary channel values, which can reproduce a broad array of colors. Pixels are the smallest individual element of an RGB image, and each pixel consists of three-channel values forming 8-bit values (range, 0–255). RGB images contain additional discriminative information compared with grayscale images (16). A very large visual information can be produced at each pixel location by varying the relative contribution of each of the three-channel values. In the direct conversion method of SWE-based ultrasomics for FLLs differentiation, we found that direct conversion of SWE images into grayscale images could change the pixel values of the original image (10). This direct conversion method may result in a mismatch between the converted image and the original color image. Therefore, we proposed a novel method that generates three single-channel (R, G, and B, respectively) grayscale images from SWE images and extracted information from every single-channel image (named as RGB three-channel method). After this decomposition, the pixel gray value of every single-channel image was consistent with that of the original RGB color image. We speculated that the RGB three-channel method could retain and reflect the same characteristics of the original color image, reducing data loss due to direct image conversion.

Few studies have reported the color image analysis method for ultrasomics features extraction in FLLs. The current study was to evaluate the diagnostic performance of the RGB three-channel method and the direct conversion method, to see whether the new method yields better results on SWE-based ultrasomics for FLLs characterization.

## Materials and Methods

This retrospective study was approved by the institutional ethics committee of our hospital, and written informed consent was obtained from all patients.

### Patients

Between January 2015 and December 2016, 127 patients with 130 liver lesions who underwent SWE examination were included in this retrospective study. The inclusion criteria were (a) distinct liver lesions larger than 10 mm at the US, (b) lesions detected with a maximum distance of 8 cm from the surface of the skin to the center of the lesion, (c) pathological confirmed lesions or clinical diagnostic standard (described in reference standard) (17). Lesions that were previously treated or relapse from previously treated, adjacent to large vessels (hepatic arterial, hepatic vein, portal vein, and the inferior vena cava) and with poor SWE image quality were excluded.

**Figure 1:**
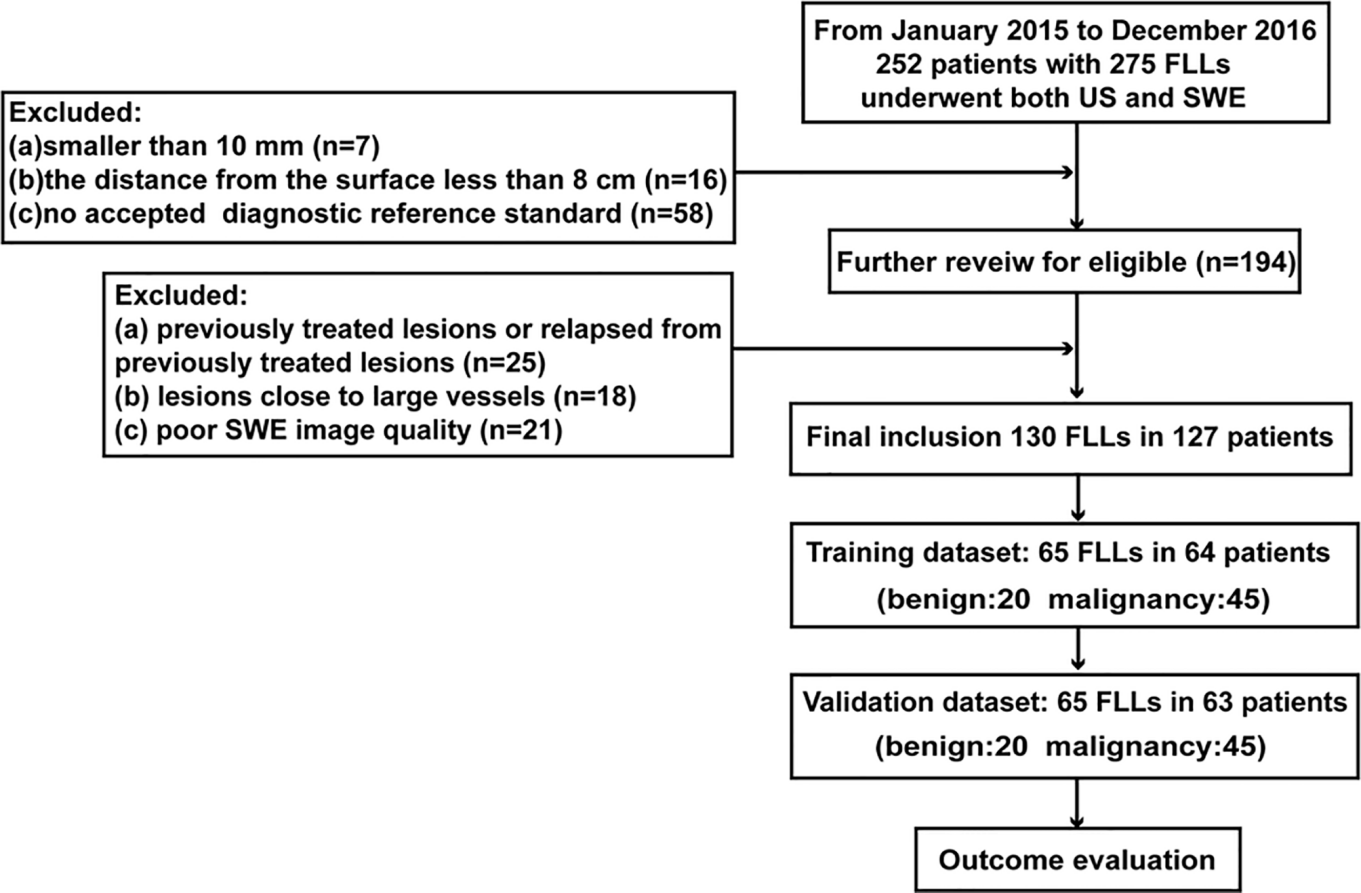
Inclusion flow chart for the study population.

Lesions were divided into the training and validation cohorts randomly in a ratio of 1:1. The inclusion flow chart for the study population is presented in **Figure 1**.

### Ultrasound Examination

SWE examinations were performed using an Aixplorer Ultrasound system (SuperSonic Imagine, Aix-en-Provence, France) equipped with the SC6-1 convex probe. One radiologist (TWS) performed the SWE examination independently according to the European Federation of Societies for Ultrasound in Medicine and Biology (EFSUMB) guidelines (18). During the examination, the patient was asked to hold their breath, and the operator should maintain immobilization for a few seconds without pressure for SWE image acquisition.

### Reference Standard

All the histopathology results of the FLLs were confirmed by either surgical resection or US-guided biopsy except for hemangiomas. As to hemangiomas, the clinical diagnosis standard was both with typical characteristics (17) on contrast-enhanced ultrasonography (CEUS) and with at least 12 months of follow-up.

### Image Conversion

**Figure 2:**
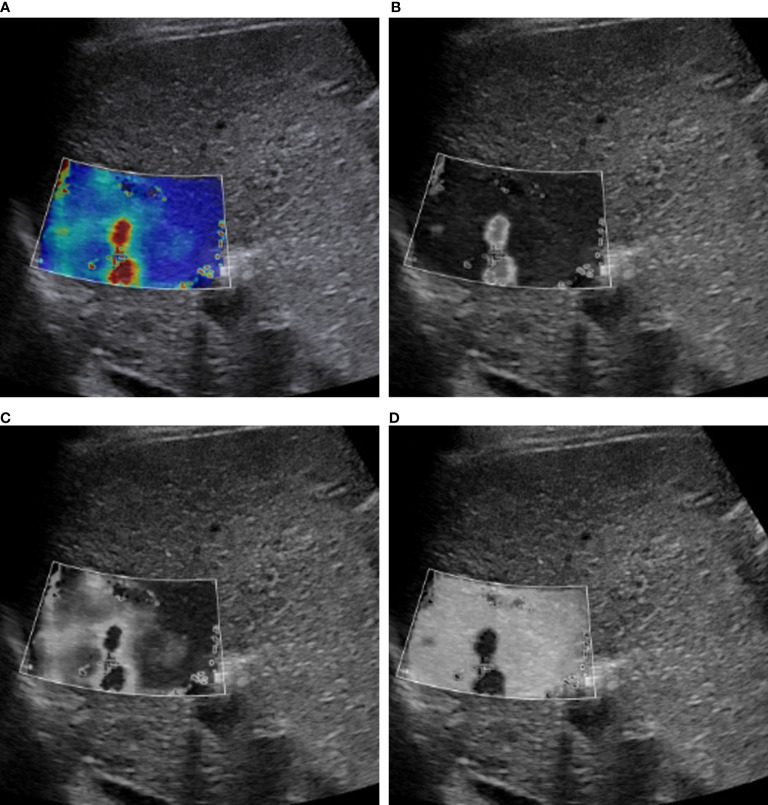
RGB image decomposition by the RGB three-channel method. A 56-year-old man with a 3.5-cm HCC in segment 2 of the liver. The depth from the body surface to the center of the lesion was <8 cm. **(A)** SWE color-code image. **(B)** Red channel. **(C)** Green channel. **(D)** Blue channel.

Image conversion was performed using Python (version 3.8.5). The direct conversion was directly converting an SWE color image into a grayscale elasticity image. The RGB three-channel method was converting an SWE color image into three single-channel images (**Figure 2**).

### Ultrasomics Score

The region of interest (ROI) was delineated along the contour of the tumor by one radiologist (WW, with more than 15 years of liver imaging experience). ROI was manually drawn from the boundary of the index mass on the grayscale image, and the same ROI was copied and pasted to the corresponding location inside the SWE image as well as every single channel image. The features were extracted from the ROI of each converted image automatically. A total of 5,936 features were extracted from every single image. In total, 17,808 features were extracted by the combination of three single-channel images generated by the RGB three-channel method, whereas 5,936 features were extracted by the direct conversion method. Most of these features were highly redundant, causing the susceptibility of the classifier. The least absolute shrinkage and selection operator (LASSO) regression was used for features reduction and selection (19). Finally, based on the selected ultrasomics features, four classifiers, namely, Adaptive Boosting (AdaBoost), Logistic Regression (LR), Support Vector Machine (SVM), and Random Forest (RF), were respectively applied to construct the ultrasomics score for FLLs characterization (4, 20, 21). The score generated by the direct conversion method and the RGB three-channel method was named direct score and RGB score, respectively.

### Technique for Oversampling

To alleviate the imbalanced medical dataset and mitigate the small data size, simple minority oversampling technique (SMOTE) (22) was applied to generate new synthetic samples at data level to create the balance between minority and majority classes.

### Model Evaluation and Comparison

The direct score model and RGB score model were applied to FLLs characterization, and the results were verified in the validation cohort.

#### Discrimination

Receiver operating curves (ROC) were used to evaluate the discrimination performance of the direct model and the RGB model in differentiating malignant from benign FLLs in the validation cohorts. It was measured by the area under the curve (AUC). To compare the predictive effect of the direct score model with the RGB score model, we calculated the net reclassification index (NRI), which was the most widely used summary statistics to present the extent of the reclassification of the models.

Calibration curves were drawn for evaluating the accurate prediction of the two models in the validation cohort. The Brier Scores were compared between two models, and a small Brier Score (23) indicates high prediction accuracy and is well-calibrated.

#### Clinical Application

A decision curve analysis (DCA) was applied to demonstrate the clinical usefulness by estimating the standardized net benefit of the prediction models at different threshold probabilities. The benefit increased with the degree of the curve deviating from the baseline.

### Statistical Analysis

Statistical analysis was performed by SPSS 21.0 for Windows (Chicago, IL), Python (version 3.8.5), R software (R Foundation for Statistical Computing, version 3.4.1; https://www.r-project.org/) and MedCalc Statistical Software version 18.5 (MedCalc Software bvba, Ostend, Belgium; http://www.medcalc.org; 2018). Chi-squared test or the Fisher exact test was used for categorical variables, and the two-sample t-test was for continuous variables in the comparison of the training and validation cohorts for baseline characteristics. Python (version 3.8.5) was applied for feature reduction and model building. LASSO regression was performed by the “sklearn” package for feature reduction. Based on “imblearn” package, SMOTE was used for upsampling and balancing the categories of training sample. The RF, SVM, adaboost, and LR classifiers were constructed by “sklearn” package. The ROC curves and the calculation of the AUC were conducted by the “pROC” package. The calibration curves and the DCA curves were plotted by the “Calibration Curves” package and “DecisionCurve” package, respectively. The R package “nricens” was used to perform NRI. A *p* < 0.05 (two-sided) was considered statistically significant.

## Results

### Patient Characteristics

One hundred twenty-seven patients with 130 lesions were enrolled in the study. Of the 130 FLLs, 90 FLLs were malignant, including hepatocellular carcinoma (n = 61), intrahepatic cholangiocarcinoma (n = 12), and liver metastasis (n = 17). Forty FLLs were benign with focal nodular hyperplasia (n = 8), hemangioma (n = 27), and inflammatory pseudotumor (n = 5). These 130 lesions were equally allocated to the training cohort and the validation cohort at random. There were 65 lesions in the training cohort including 45 malignant lesions and 20 benign lesions. After simple minority oversampling, the training cohort changed into 90 lesions with 45 malignancy and 45 benign lesions. The baseline characteristics of the two cohorts were compared in **Table 1**.

**Table 1:** Clinical–pathological characteristics and ultrasomics score in the training and validation cohorts.

	Training cohort	Validation cohort	*p* value
**By patient**			
Gender (male/female)	45/19	45/18	1.000
Age (mean ± SD)*	51.3 ± 13.8	47.0 ± 13.8	0.077
Hepatitis (positive/negative)	29/35	33/30	0.480
AFP (<20/≥20) (ng/ml)	43/21	42/21	1.000
**By lesion**			
Lesion size (mean ± SD) * (cm)	5.3 ± 3.2	5.6 ± 3.2	0.520
Pathology (benign/malignant)	20/45	20/45	1.000
Benign			
Hemangiomas	15/20	12/20	0.501
FNH	2/20	6/20	0.235
Inflammatory pseudotumor	3/20	2/20	1.000
Malignant			
HCC	31/45	30/45	1.000
ICC	6/45	6/45	1.000
MLC	8/45	9/45	1.000
Ultrasomics score*			
Direct score	0.66 ± 0.40	0.64 ± 0.23	0.686
RGB score	0.68 ± 0.41	0.63 ± 0.32	0.452

Unless otherwise indicated, data are numbers.

*Data are mean ± standard deviation.

AFP, alpha-fetoprotein; RGB, red, green and blue; FNH, focal nodular hyperplasia; HCC, hepatocellular carcinoma; ICC, intrahepatic cholangiocarcinoma; MLC, metastatic liver cancer.

Direct score refers to the application of direct conversion method to the image of the cases in the training and validation sets, and obtain the risks core of each case. RGB score refers to the application of RGB three-channel conversion method to the image of the cases in the training and validation sets, and obtain the risks core of each case.

#### Feature Selection and Analysis of Ultrasomics

Of 130 FLLs, 30.8% (40/130) were benign and 69.2% (90/130) were malignant. Based on the two conversion methods, 5,936 features were extracted from the direct conversion method, whereas 17,808 features were extracted from the RGB three-channel method. After ultrasomics feature selection by LASSO regression, 29 features from the direct conversion method and 8 features from the RGB three-channel method were potential predictors for differentiating FLLs. The extracted features for model construction and the definitions of the features are presented in **Supplementary Material**.

### Comparison of Two Ultrasomics Models

**Figure 3:**
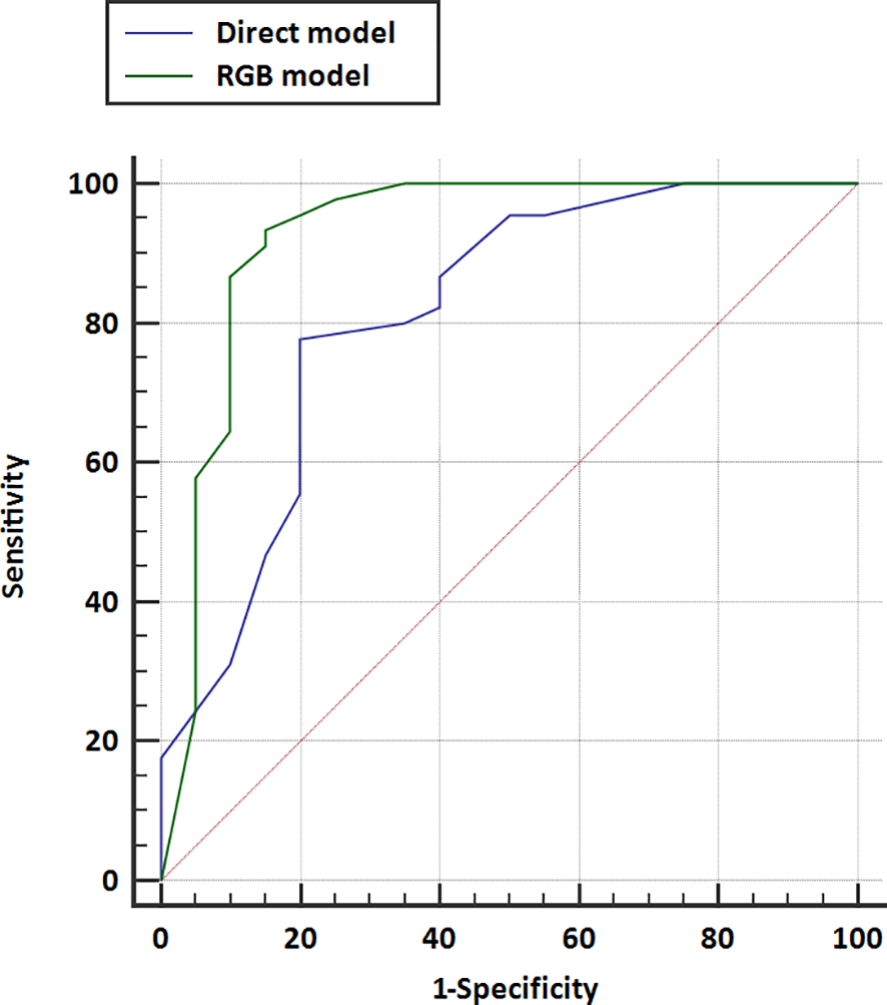
Receiver operating characteristic (ROC) curves for the direct model and RGB model were performed in the validation cohort. The area under the curve for direct model US and RGB model were 0.813 (95% CI, 0.697–0.899) and 0.926 (95% CI, 0.833–0.976), respectively.

The AUCs of models using different classifiers are list in **Table 2**. The results showed that RF (AUC = 0.813 and 0.926 for direct model and RGB model, respectively) outperformed the other classifiers. RF has stability and effectiveness with high staging performance. The diagnostic sensitivity, specificity, and accuracy of the direct model using RF as a classifier were 86.7% (95% CI, 73.2%, 94.9%), 60.0% (95% CI, 36.1%, 80.9%), and 78.5% (95% CI, 58.4%, 103.2%), respectively, while those of the RGB model were 93.3% (95% CI, 81.7%, 98.6%), 85% (95% CI, 62.1%, 97.8%), and 90.8% (95% CI, 69.1%, 117.1%). The ROC curves were used to demonstrate the prediction accuracy of the two ultrasomics models in the validation cohort. The AUCs of the RGB model and the direct model for FLLs characterization were 0.926 (95% CI, 0.833–0.976) and 0.813 (95% CI, 0.697–0.899), respectively (*p* = 0.038) (**Table 2**), indicating that the RGB model was more effective in FLLs characterization (**Figure 3**). NRI was calculated for quantifying improvement in predicting the accuracy of the two ultrasomics models. NRI revealed that the RGB model exhibited a better reclassification in the FLLs discrimination. 7% of malignant lesions and 25% of benign ones were reclassified accurately in comparison with the direct model (*p* = 0.01) (**Table 3**).

**Table 2:** Diagnostic performance of direct model and RGB model among different classifiers in the validation cohort.

Model		Cutoff value^†^	Sensitivity	Specificity	Accuracy	PPV	NPV
			(95% CI)	(95% CI)	(95% CI)	(95% CI)	(95% CI)
**RF**	**Direct**	0.500	0.867	0.600	0.785	0.830	0.667
			(0.732, 0.949)	(0.361, 0.809)	(0.584, 1.032)	(0.738, 0.894)	(0.467, 0.820)
	**RGB**	0.450	0.933	0.850	0.908	0.933	0.850
			(0.817, 0.986)	(0.621, 0.978)	(0.691, 1.171)	(0.831, 0.976)	(0.652, 0.945)
**SVM**	**Direct**	0.701	0.822	0.500	0.723	0.787	0.556
			(0.679, 0.920)	(0.272, 0.728)	(0.531, 0.962)	(0.700, 0.854)	(0.368, 0.729)
	**RGB**	0.661	0.756	0.850	0.785	0.919	0.607
			(0.605, 0.871)	(0.621, 0.968)	(0.584, 1.032)	(0.798, 0.970)	(0.472, 0.727)
**AdaBoost**	**Direct**	0.585	0.578	0.800	0.646	0.867	0.457
			(0.422, 0.723)	(0.563, 0.943)	(0.466, 0.873)	(0.723, 0.942)	(0.359, 0.558)
	**RGB**	0.531	0.800	0.850	0.815	0.923	0.654
			(0.654, 0.904)	(0.621, 0.968)	(0.611, 1.067)	(0.807, 0.972)	(0.506, 0.777)
**LR**	**Direct**	0.273	0.822	0.450	0.708	0.771	0.529
			(0.679, 0.920)	(0.231, 0.685)	(0.518, 0.944)	(0.689, 0.836)	(0.337, 0.713)
	**RGB**	0.686	0.711	0.900	0.769	0.941	0.581
			(0.557, 0.836)	(0.683, 0.988)	(0.571, 1.014)	(0.809, 0.984)	(0.461, 0.691)

Unless otherwise indicated, data are percentages, and data in parentheses are 95% confidence intervals.

^†^Data are cutoff risk score (the output is the specific value, 0–1).

CI, confidence interval; PPV, positive predictive value; NPV, negative predictive value; RGB, red, green, and blue; RF, random forest; SVM, support vector machine; AdaBoost, adaptive boosting; LR logistic regression.

Direct model refers to the model construction based on the application of direct conversion method to the image of the cases in validation set. RGB model refers to the model construction based on the application of RGB three-channel conversion method to the image of the cases in the validation set.

**Table 3:** Evaluation the performance of direct model and RGB model of the validation cohort.

Models		AUC		NRI			
		AUC (95% CI)	*p*-value	NRI+	NRI−	NRI	*p*-value
**RF**	**Direct**	0.813 (0.697, 0.899)	*p* = 0.038	–	–	–	*p* = 0.010
	**RGB**	0.926 (0.833, 0.976)		0.067	0.250	0.317	
**SVM**	**Direct**	0.660 (0.532, 0.773)	*p* = 0.010	–	–	–	*p* = 0.084
	**RGB**	0.857 (0.748, 0.931)		−0.067	0.350	0.283	
**AdaBoost**	**Direct**	0.679 (0.551, 0.789)	*p* = 0.002	–	–	–	*p* = 0.005
	**RGB**	0864 (0.757, 0.937)		0.222	0.050	0.272	
**LR**	**Direct**	0.571 (0.442, 0.693)	*p* = 0.001	–	–	–	*p* = 0.043
	**RGB**	0.870 (0.763, 0.941)		−0.111	0.450	0.339	

Data are percentages and data in parentheses are 95% confidence intervals.

AUC, area under the curve; CI, confidence interval; RF, random forest; SVM, support vector machine; AdaBoost adaptive boosting; LR, logistic regression; NRI+, movement in predicted risks introduced by change of models in malignant cases; NRI−, movement in predicted risks introduced by changes of model in benign cases.

Direct model refers to the model construction based on the application of direct conversion method to the image of the cases in validation set. RGB model refers to the model construction based on the application of RGB three-channel conversion method to the image of the cases in the validation set.

#### Calibration

**Figure 4:**
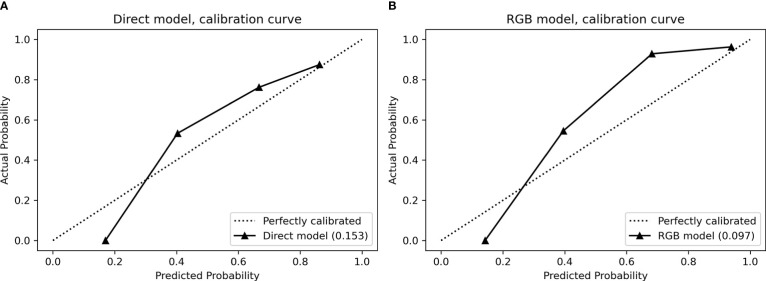
Calibration curves for **(A)** direct model and **(B)** RGB model performed in the validation cohort. The calibration curves demonstrated a statistical goodness-of-fit measurement of the models in the characterization of focal liver lesions. The solid line represented the performance of the models, and the dotted line represented an ideal model. The lesser the solid line deviated from the dotted line, the better the calibration of the model.

In the validation cohort, the calibration curves were drawn to explore whether the predicted probability was well-agreed with the real probability in the direct model (**Figure 4A**) and RGB model (**Figure 4B**). The reliability of the calibration curves was assessed by the Brier Scores. A small Brier Score indicated high prediction accuracy and well calibration. The Brier Scores of the direct model and RGB model were 0.153 and 0.097, respectively, indicating the RGB model had a higher prediction accuracy and better agreement for the calibration curves compared with the direct model.

#### Clinical Application

**Figure 5:**
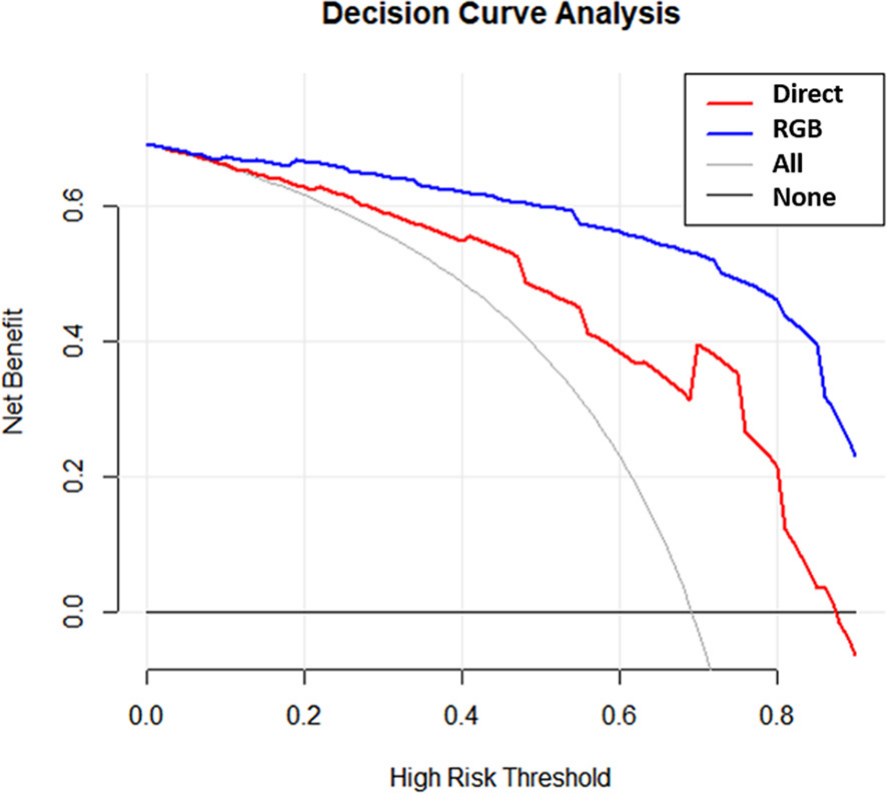
Decision curves for the direct model and RGB model were performed in the validation cohort.

DCAs of the direct score model and the RGB score model were compared in the validation cohort (**Figure 5**). The DCA curves revealed that at any given threshold probability, the RGB score model in predicting malignancy had a greater net benefit compared with that of the direct score model.

## Discussion

This retrospective study proposed a new RGB three-channel method for SWE-based ultrasomics color image analysis. The diagnostic performance of the RGB model generated by this method was tested in the validation cohort regarding discrimination, calibration, and clinical application. The RGB model exhibited superior diagnosis performance than the direct model, with AUC of 0.813 for direct model and 0.926 for RGB model. The RGB model outperformed the direct model concerning prediction accuracy, calibration, and clinical application. Images converted from the RGB three-channel method could well reflect the original information of the RGB color image, which reduced less data loss compared with the direct conversion method. This method captured more information and may be helpful in clinical practice.

Recently, the most common color image processing was directly converted into a grayscale image, which was based on the gray intensity = 0.2126R + 0.7152G + 0.0722B. This method is easy to apply, but the most important limitation is color information lost during the convention (10). To seek an easy and effective way on color image analysis for radiomics or ultrasomics, several research groups have proposed different methods for color image processing (5, 11–15, 24). Bhatia et al. (11) separated the color components of the 2D-SWE image and generated a pure color-code image from removing the layer of the grayscale image. They established a prediction model in the identification between benign and malignant thyroid nodules by extracting 15 gray level co-occurrence matrix features (GLCM). The sensitivity, specificity, and AUC of this model reached 97.5%, and 90%, and 0.973, respectively. Gatos et al. (13, 14) developed an RGB-to-stiffness inverse mapping technique in assessing liver stiffness. This method presented excellent results in the evaluation of liver stiffness with an ACU of 0.87. The above methods required intermediate image processing step for color to grayscale conversion. Ma et al. (15) used a histogram-based color image analysis method in positron emission tomography computed tomography (PET-CT) image in the differentiation of nonsmall cell lung carcinoma subtypes. The drawback of this method was that information about the object’s location, shape, and texture was discarded. In their study, they combined texture and color features and got an AUC of 0.89 (95% CI, 0.78–1.00). Yao et al. (24) established a prediction model by extracting features of multimodal ultrasound images in FLLs characterization. The feature extraction method used in this study was built on sparse representation theory (SRT), which was different from the traditional Radiomics method. Based on this SRT method, color image analysis was more effective without the need to convert into grayscale images that occur in traditional radiomics analysis. However, the image processing and feature extraction process in their study required highly specialized skills in computer programming, which was hard to conduct in clinical applications. In this study, the RGB three-channel method was easier to implement than the above image processing methods, and according to our results, the RGB three-channel method can achieve a good diagnostic performance.

The RGB three channels in the SWE color image were mainly used to reflect the stiffness distribution of the lesion, from the lowest stiffness (dark blue) to the highest stiffness (red) and in between is green (5, 25). This study indicated the RGB three-channel method could better retain the original information without data loss compared with the direct conversion method, which was important for subsequent data mining. We hypothesized this reason may be due to the change of grayscale pixel value after direct conversion, consequently resulting in loss of color image data. In the RGB three-channel method, the original color image was converted into three single-channel grayscale images. Each grayscale image reflects the pixel values of each color component in the original color image, and the grayscale pixel value remained unchanged compared with the original color image.

Medical image analysis is based on the different grayscale values and textures between the lesion and normal tissue with the concept that image information can reveal the relation between underlying pathophysiology and quantitative features (8). However, color image analysis is also important in radiomics and ultrasomics. It can provide additional information for lesion characterization. SWE is a color-coded image with red, green, and blue reflecting the difference in stiffness according to the different propagation speeds of the shear wave in tissue. PET-CT image is a color-code image that can reflect metabolic information of the tissues by detecting the distribution of increased tracer uptake values (26). The brightness of color indicated the degree of tracer aggregation. In this study, we presented an RGB three-channel method for color image analysis on ultrasomics, which can retain as much information as the original color image. This method was easy to access with great potential for clinical application.

There were some drawbacks and limitations to this study. First, our study was limited by a small patient population, which may lead to overfitting and model instability. Second, our data were retrospectively collected from a single institution, which may limit generalizability to populations in other geographical regions. Besides, ROIs for features extraction were drawn manually, and the interobserver reproducibility was not evaluated. Finally, we did not apply deep learning networks in this study as presented in Gatos et al. (27) and Kagadis et al. (28) studies. Further studies with a larger sample size and the application of deep learning networks would be taken to assess the diagnosis performance of this new method for widespread implementation in clinical practice.

## Conclusion

In conclusion, the RGB three-channel method for SWE-based ultrasomics analysis can effectively retain the original image information and improve the diagnostic performance in differentiating FLLs. Our research provides a new technique for how to better process ultrasomics analysis of color images and expand the clinical application of ultrasomics on color image analysis.

## Data Availability Statement

The raw data supporting the conclusions of this article will be made available by the authors, without undue reservation.

## Ethics Statement

The studies involving human participants were reviewed and approved by The First Affiliated Hospital of Sun Yat-sen University. The patients/participants provided their written informed consent to participate in this study.

## Author Contributions

Guarantors of integrity of entire study: M-QC, W-ST, M-DLi, H-TH, S-MR, and L-DC. Study concepts/study design or data acquisition or data analysis/interpretation: all authors. Manuscript drafting or manuscript revision for important intellectual content: all authors. Agrees to ensure any questions related to the work are appropriately resolved: all authors. Literature research: M-QC, W-ST, M-DLi, S-MR, and L-DC. Clinical studies: M-QC, W-ST, M-DLi, H-TH, WL, S-MR, and L-DC. Statistical analysis: M-QC, W-ST, J-CZ, YH, S-MR, and L-DC. Manuscript editing: M-QC, W-ST, X-YX, M-DLu, MK, WW, S-MR, and L-DC. Revison of the manuscript: M-FX and M-QC. All authors contributed to the article and approved the submitted version.

## Funding

This work was supported by the National Nature Science Foundation of China (No: 81971630) and the Guangzhou Science and Technology Foundation (No: 201904010187).

## Conflict of Interest

The authors declare that the research was conducted in the absence of any commercial or financial relationships that could be construed as a potential conflict of interest.

## Publisher’s Note

All claims expressed in this article are solely those of the authors and do not necessarily represent those of their affiliated organizations, or those of the publisher, the editors and the reviewers. Any product that may be evaluated in this article, or claim that may be made by its manufacturer, is not guaranteed or endorsed by the publisher.

## Abbreviations

AUC, area under the curve; SWE, shear wave elastography; NRI, net reclassification index; DCA, decision curve analysis; LASSO, least absolute shrinkage and selection operator; ROC, receiver operating characteristic; ROI, region of interest; RGB, red, green, and blue; CI, confidence interval; FLLs, focal liver lesions; PET-CT, positron emission tomography computed tomography; SRT, sparse representation theory; AdaBoost, adaptive boosting; SVM, support vector machine; RF, random forest; LR, logistic regression.
