# Supplementary material for: RGB Three-Channel SWE-Based Ultrasomics Model: Improving the Efficiency in Differentiating Focal Liver Lesions
Source: Front Oncol. 2021 Sep 27;11:704218. doi: 10.3389/fonc.2021.704218 (PMC8504873; doi:10.3389/fonc.2021.704218)
Supplement: Supplementary file 1 [file DataSheet_1.docx]

**Extracted features for direct model**

According to the direct model, 5936 extracted features were reduced to 29 potential predictors using LASSO regression, including:

original_firstorder_Kurtosis

CoLIAGe2D_WindowSize7_Sum Average_firstorder_Skewness

CoLIAGe2D_WindowSize11_Sum Average_firstorder_RootMeanSquared

wavelet-LLL_lbp-3D-m2_firstorder_Skewness

shearlet2DIdxs[ 1 1 -2]_ngtdm_Contrast

shearlet2DIdxs[1 1 0]_firstorder_Kurtosis

shearlet2DIdxs[ 1 2 -2]_glszm_SmallAreaEmphasis

shearlet2DIdxs[1 2 0]_firstorder_Kurtosis

shearlet2DIdxs[ 1 3 -4]_glszm_SmallAreaEmphasis

shearlet2DIdxs[ 1 3 -2]_glszm_SmallAreaHighGrayLevelEmphasis

shearlet2DIdxs[1 3 0]_firstorder_Uniformity

shearlet2DIdxs[1 3 0]_glszm_GrayLevelVariance

shearlet2DIdxs[2 2 0]_glszm_SmallAreaEmphasis

shearlet2DIdxs[ 2 3 -3]_glszm_LowGrayLevelZoneEmphasis

shearlet2DIdxs[ 2 3 -1]_glcm_Autocorrelation

shearlet2DIdxs[2 3 0]_glszm_SmallAreaLowGrayLevelEmphasis

shearlet2DIdxs[2 3 2]_glszm_LargeAreaHighGrayLevelEmphasis

glbp_hist_kernel7_2

gldp_hist_0_kernel1_8

gldp_hist_45_kernel7_1

gldp_hist_45_kernel7_3

gldp_hist_90_kernel11_4

gLTCoPs1_hist_kernel0_3

gLTCoPs1_hist_kernel7_3

gltp1_hist_kernel1_3

gltp1_hist_kernel3_1

PLBP_hist_tumor_orient2_0

PLBP_hist_tumor_orient2_9

PLBP_hist_tumor_orient7_9

The features were concluded into 5 kinds and their definition are listed as follows:

1. Original_first-order: The distribution of voxel intensities within the image region defined by the mask.

2. CoLIAGe: Co-occurrence of local anisotropic gradient orientations, features which were captures higher order co-occurrence patterns of local gradient tensors at a pixel/voxel level to distinguish disease phenotypes that have similar morphologic appearances.

3. Wavelet: The 2D-DWT reflects most of the edge information of the image, including the horizontal and vertical directions of the image and the high-frequency parts in both directions.

4. Shearlet: Shearlet capture the curvilinear geometry structure in the image and is suitable

5. Gabors_gldp: LDP extracts image edge information and gradient information, containing more detailed discriminative features as compared to LBP.

6. Gabors_gltp: LTP is an extension of LBP and has good robustness to light change and noise interference.

7. PLBP: PLBP is an oriented local texture descriptor that combines the phase congruency (PC) approach with the local binary pattern (LBP).

**Extracted features for RGB model**

According to the RGB model, 17808 extracted features were reduced to 8 potential predictors using the LASSO regression, including:

original_glcm_Correlation_r

textural_phenotype_level_60-70%_r

gLTCoPs1_hist_kernel0_3_r

CoLIAGe2D_WindowSize7_Sum Average_firstorder_Skewness_g

shearlet2DIdxs[1 1 -2]_gldm_DependenceNonUniformityNormalized_g

shearlet2DIdxs[1 2 0]_firstorder_Kurtosis_g

shearlet2DIdxs[2 3 0]_gldm_DependenceVariance_g

gLTCoPs1_hist_kernel0_3_g

The features were concluded into 5 kinds and their definition are listed as follows:

1. Original_ GLCM : The grey level co-occurrence matrix, features which calculated how often pairs of pixels with specific values and in a specified spatial relationship occur in an image. The second-order joint probability function of an image region constrained by the mask.

2. Textural _phenotype: Features capture the transition in textural appearance.

3. Gabors_ gLTCoPs: The LTCoP encodes the co-occurrence of similar ternary edges which are calculated based on the gray values of center pixel and its surrounding neighbors, being robust to the different lighting conditions as compared to LBP and LDP.

4. Shearlet: Shearlet capture the curvilinear geometry structure in the image and is suitable for processing high-dimensional signals.

5. CoLIAGe: Co-occurrence of local anisotropic gradient orientations, features which were captures higher order co-occurrence patterns of local gradient tensors at a pixel/voxel level to distinguish disease phenotypes that have similar morphologic appearances.
